# Gender differences in alcohol-related non-consensual sex; cross-sectional analysis of a student population

**DOI:** 10.1186/1471-2458-12-216

**Published:** 2012-03-20

**Authors:** Clare Gunby, Anna Carline, Mark A Bellis, Caryl Beynon

**Affiliations:** 1 School of Law, Liverpool John Moores University, John Foster Building, 98 Mount Pleasant, Liverpool L3 5UZ, UK; 2 Centre for Public Health, Liverpool John Moores University, Henry Cotton Building, 15-21 Webster Street, Liverpool L3 2ET, UK

**Keywords:** Alcohol, Sex, Sexual assault, Law, Gender, AUDIT, Violence

## Abstract

**Background:**

Sexual offences are a global public health concern. Recent changes in the law in England and Wales have dramatically altered the legal landscape of sexual offences, but sexual assaults where the victim is voluntarily intoxicated by alcohol continue to have low conviction rates. Worldwide, students are high consumers of alcohol. This research aimed to compare male and female students in relation to their knowledge and attitudes about alcohol and sexual activity and to identify factors associated with being the victim of alcohol-related non-consensual sex.

**Methods:**

1,110 students completed an online questionnaire. Drinking levels were measured using the Alcohol Use Disorder Identification Test. Non-consensual sexual experiences were measured using the Sexual Experience Survey. Univariate and multivariate analyses were undertaken using chi square and backwards stepwise logistic regression respectively.

**Results:**

A third of respondents had experienced alcohol-related non-consensual sex. Male and female students differed in the importance they gave to cues in deciding if a person wished to have sex with them and their understanding of the law of consent. 82.2% of women who had experienced alcohol-related non-consensual sex were hazardous drinkers compared to 62.9% who drank at lower levels (P < 0.001). Differences existed between men and women, and between those who had and had not experienced alcohol-related non-consensual sex, in relation to assessments of culpability in scenarios depicting alcohol-related intercourse. A third of respondents believed that a significant proportion of rapes were false allegations; significantly more men than women responded in this way.

**Conclusions:**

Alcohol-related coerced sexual activity is a significant occurrence among students; attitudinal and knowledge differences between males and females may explain this. Educational messages that focus upon what is deemed acceptable sexual behaviour, the law and rape myths are needed but are set against a backdrop where drunkenness is commonplace.

## Background

Sexual offences are a public health problem worldwide and impact upon people of all ages and from all social groupings [1,2]. Scholars argue that rape is one of the most prevalent, yet least recognised, human rights issues in the world [3]. Women in England and Wales fear being the victim of rape more than any other offence [4]. Victims of rape form the largest proportion of people suffering from Post Traumatic Stress Disorder which is associated with a range of outcomes including feelings of anger, shame and denial, relationship difficulties, substance dependence and increased levels of depression and suicide [5,6]. The physical consequences of sex crimes include injury, sexually transmitted infections and unwanted pregnancies [7].

In recent years there have been significant changes to the way that sexual offences are dealt with in jurisdictions across the western world. In England and Wales, advancements include the introduction of special measures to assist victims to give evidence in court, improved practices and protocols for working with survivors and an expansion of services to support victims [2]. Furthermore, a review of the law in relation to sexual offences culminated in the Sexual Offences Act (2003); legislation that dramatically altered the legal landscape relating to sexual offences generally and the offence of rape specifically. Despite these amendments concerns around the rape conviction rate remain [8-11]. Alleged sexual assaults are particularly difficult to prosecute when the victim is voluntarily intoxicated by alcohol at the time of the incident [2,12].

In the UK, alcohol consumption is a recognised risk factor for experiencing a sexual offence, and Police data indicate that in around half of rape cases the complainant had been drinking [13]. However, the contribution of alcohol in sexual offences is somewhat confused by societal views on the role alcohol plays in sexual situations. Western society is permeated with positive images linking alcohol and sex [14], while research demonstrates that alcohol is often used, especially by young people, to facilitate sexual encounters and produce sexual effects [15,16]. As sex crimes often occur following social interactions involving alcohol consumption [17] it is realistic to surmise that non-consensual sex occurs, in certain cases, when consensual sex is also a potential outcome. Consequently, a person's interpretation of the sexual situation may influence the potential for assaultive behaviour. Indeed, in order to mitigate possible rejection, the cues men and women use to signify attraction are typically ambiguous [18]. This can easily lead to misinterpretation which is likely to be exacerbated when alcohol disrupts cognitive processes making it more difficult to evaluate complex stimuli and situations [19]. Differences between men and women in relation to attitudes and knowledge of sexual situations are also likely to influence the outcome of such occurrences [20-24].

There is much evidence to show the association between alcohol use and non-consensual sex in American student populations. In a recent study of 2,000 women, 6.4% reported that they had been the victim of a rape where drugs and/or alcohol had resulted in incapacitation [25]. In 96% of these cases alcohol was identified as the substance used to procure sex and in the majority of instances alcohol had been consumed voluntarily. There has been little research with students in England about their experiences of alcohol-related sexual assault which is perhaps surprising when alcohol consumption among this population is perceived to be high [26,27].

The first aim of this study was to compare male and female students' attitudes and knowledge about alcohol and sexual activity and their drinking behaviour. More specifically, we compared male and female students' knowledge of the laws of consent; attitudes towards the cues they deemed important in deciding if another person would agree to have sex with them; attitudes towards the effects of alcohol on a person's ability to consent to sex; attitudes about the impact of alcohol on the reporting of rape; and attitudes on the culpability or otherwise of women who are raped when they have been drinking alcohol. The second aim was to identify those factors that were associated with male and female students being the victim of alcohol-related non-consensual sex; again, a comparison was made in terms of their drinking behaviour and attitudes and knowledge about alcohol and sexual activity. Students aged 18 to 24 were included in the study because people aged 16 to 24 have the highest risk of experiencing non-consensual sex [28-30], while 18 represents the minimum legal age for purchasing alcohol in the United Kingdom.

## Methods

One hundred and ten students aged 18 to 24 years were recruited to the study using non-probability sampling from a single university in the North West of England. These students completed an anonymous online questionnaire from October 2008 to January 2009. Undergraduate and postgraduate students within four Faculties received an email invitation to participate in the questionnaire, available through a link embedded in the email. Posters detailing the study and inviting students to participate were also displayed in student areas of the university. This recruitment strategy meant that a small number of people outside of the university completed the questionnaire; of these 22 were students at other universities who met the age criteria and their responses were included.

The Sexual Experience Survey (SES), developed in the USA in the late 1970s, is the best available measure of non-consensual sexual experiences [31] and was used to identify alcohol-related non-consensual sex that occurred since the age of 14. The SES includes features that are widely recognised as standard approaches to the assessment of sexual victimisation. An example is the avoidance of the terms 'rape' and 'sexual assault' which participants frequently fail to respond to because they do not label their experiences as such, even if their experiences meet the legal definition for such offences. Instead the SES uses behaviourally specific descriptions of acts and tactics that mirror legal definitions of specific sexual offences as follows: 1) serving me high alcohol content drinks when they appeared to be regular strength drinks until I was too intoxicated (drunk) to give consent or stop what was happening; 2) using me sexually when I was asleep or unconscious from alcohol and when I came round (gained consciousness) I could not give consent or stop what was happening; 3) encouraging or pressurising me to drink alcohol until I was too intoxicated (drunk) to give consent or stop what was happening; and 4) using me sexually after I had been drinking alcohol and was conscious but too intoxicated (drunk) to give consent or stop what was happening. Participants were asked how many times they had experienced each of these four scenarios in relation to oral sex, vaginal penetration (by the penis, finger or other objects) and anal penetration (by the penis, fingers or other objects). An internal consistency reliability of 0.74 has been reported for the SES for female victims and test-retest agreement rates over a one week administration period were reported to be 93% consistent [32]. Since its development the SES has been revised in order to reflect changes in the law and the strategies used by perpetrators to procure sex. The most recent revisions occurred in 2006 during which time the questions became gender neutral [33]. Following discussions with the SES author the term 'butt' was substituted with 'anus' in order to make the questions relevant to a UK population. Responses from the SES questions were re-coded into a single dichotomous variable for whether or not the respondent had experienced alcohol-related non-consensual sex, be it oral, vaginal or anal since the age of 14.

Drinking levels were measured using the Alcohol Use Disorder Identification Test (AUDIT), a tool developed by the World Health Organization to facilitate screening of excessive drinking [34]. AUDIT has a high internal consistency reliability of 0.84 [35] and high test-retest reliability [36]. The five item AUDIT has been recommended for use with student populations [37] and was therefore utilised here. Each item is scored zero to four giving a total of 20 and scores of five and above are taken as an indicator of hazardous drinking [37].

Other sections of the questionnaire included demographic details and questions on: knowledge about the legal definition of consent; attitudes held by students on an individual's capacity to consent to sex when alcohol had been consumed; attitudes towards the legality of scenarios depicting sex between intoxicated people; and attitudes towards alcohol-related sex, alcohol-related rape, false rape allegations and the role of alcohol in such circumstances. The questionnaire also described nine different actions or circumstances and participants were asked to indicate how relevant each cue was in helping to decide if the other person would agree to have sex with them. These cues were: 1) if the other person has been flirting with you during the evening; 2) if the other person has been kissing you during the evening; 3) if the other person has voluntarily removed some of their clothing for you; 4) if the other person has voluntarily removed some of your clothing; 5) if the other person accepted a drink from you during the evening; 6) if the other person verbally agrees to have sex with you; 7) if you've had sex with the other person previously; 8) if the other person has a reputation for sleeping around; and 9) if the other person has agreed to go back to your house.

All questions were gender neutral and devised following a review of the related literature. A pilot study was conducted with twelve students aged 18 to 24 years attending an East Midlands university and amendments were made accordingly. The research received approval from Liverpool John Moores University's ethics committee.

Univariate comparisons for male versus female, and those that had, versus those who had not, experienced alcohol-related non-consensual sex were conducted using chi square analyses because data were categorical. Backwards stepwise logistic regression models with simple contrasts were used to examine multivariate associations. Only records with complete data were included in multivariate analyses to ensure that likelihood ratio tests compared nested models. Analysis was conducted using SPSS v17 [38] and significance was set at P < 0.05 for all tests.

## Results

The questionnaire data were investigated for erroneous and missing values. Of the 1,110 participants that completed the questionnaire, 31 (2.8%) were removed because they did not state their age or that they were a student. Of the remaining 1,079 respondents, 817 (75.8%) were female, 259 (24.0%) were male and two (0.2%) identified themselves as transgender. The two transgender respondents were removed from subsequent analyses by sex because the sample size was too small to make this viable. The majority of respondents were white British (N = 902, 83.8%). Participants' ages were as follows: 393 (36.4%) were aged 18 to 19 years, 451 (41.8%) were aged 20 to 21 years, 167 (15.5%) were aged 22 to 23 years and 68 (6.3%) were aged 24 years. Of the 1,072 people who responded to the SES questions, 329 (30.7%) had experienced alcohol-related non-consensual sex since the age of 14; this included 55 men and one person who was transgender.

Bivariate analyses revealed a number of differences between men and women in relation to their knowledge and attitudes to alcohol and sexual activity (Table 1). A number of these differences persisted in the multivariate analysis (Table 2). When asked which cues were important when deciding if another person wanted to have sex with them, 59.1% of men and 45.0% of women felt that the other person flirting with them was relevant compared to 22.4% of men and 39.9% of women who felt this was irrelevant (adjusted odds ratio [AOR] = 1.83, 95% confidence interval [CI] = 1.24-2.71). Similarly, 90.3% of men reported that another person removing some of the respondent's clothes was relevant to them deciding if that person wanted to have sex with them compared to 3.1% who felt this was irrelevant. Comparable figures for women were 74.2% and 14.6% respectively (AOR = 3.04, 95% CI = 1.15-8.00). While the majority of participants (90.0%) knew that consent involved agreeing to engage in sex through choice, men were significantly less likely to know this is the case than women (AOR = 0.30, 95% CI = 0.13-0.69) or were unsure whether this was the case (AOR = 0.32, 95% CI = 0.11-0.90). Approximately half of respondents thought that consent needed to be verbally agreed; a larger proportion of women than men incorrectly believed this was the law (AOR = 0.63, 95% CI = 0.44-0.90). Men were less likely to agree that being drunk affects one's capacity to consent to sex than to disagree (AOR = 053, 95% CI = 0.35-0.79).

**Table 1:** Bivariate comparison of male and female students' alcohol consumption and sexual activity

Variable	Category	Total	Female	Male		
		**N (%)**	**N (%)**	**N (%)**	***X*^2^**	**P**

AUDIT score	Non-hazardous	305 (28.8)	246 (30.6)	59 (23.2)		
	
	Hazardous	753 (71.2)	558 (69.4)	195 (76.8)	5.11	0.024

Relevance of other person flirting with you when deciding whether they will agree to have sex with you	Irrelevant	384 (35.7)	326 (39.9)	58 (22.4)		
	
	Undecided	171 (15.9)	123 (15.1)	48 (18.5)		
	
	Relevant	521 (48.4)	368 (45.0)	153 (59.1)	26.38	< 0.001

Relevance of the other person kissing you when deciding whether they will agree to have sex with you	Irrelevant	212 (19.8)	192 (23.6)	20 (7.8)		
	
	Undecided	116 (10.8)	90 (11.0)	26 (10.1)		
	
	Relevant	745 (69.4)	533 (65.4)	212 (82.2)	32.89	< 0.001

Relevance of the other person removing some of their clothes when deciding whether they will agree to have sex with you	Irrelevant	128 (12.0)	118 (14.6)	10 (3.9)		
	
	Undecided	120 (11.2)	105 (13.0)	15 (5.8)		
	
	Relevant	821 (76.8)	587 (72.5)	234 (90.3)	35.95	< 0.001

Relevance of the other person removing some of your clothes when deciding whether they will agree to have sex with you	Irrelevant	126 (11.8)	118 (14.6)	8 (3.1)		
	
	Undecided	108 (10.1)	91 (11.2)	17 (6.6)		
	
	Relevant	832 (78.0)	600 (74.2)	232 (90.3)	32.34	< 0.001

Relevance of the other person verbally agreeing to have sex with you when deciding whether they will agree to have sex with you	Irrelevant	55 (5.1)	49 (6.0)	6 (2.3)		
	
	Undecided	51 (4.8)	42 (5.2)	9 (3.5)		
	
	Relevant	964 (90.1)	722 (88.8)	242 (94.2)	6.94	0.031

Relevance of the other person agreeing to go back to your house when deciding whether they will agree to have sex with you	Irrelevant	356 (33.1)	289 (35.4)	67 (26.0)		
	
	Undecided	235 (21.9)	184 (22.5)	51 (19.8)		
	
	Relevant	483 (45.0)	343 (42.0)	140 (54.3)	12.49	0.002

Consent is about agreeing to sex through choice	No	31 (2.9)	17 (2.1)	14 (5.4)		
	
	Unsure	76 (7.1)	61 (7.5)	15 (5.8)		
	
	Yes	961 (90.0)	733 (90.4)	228 (88.7)	8.39	0.015

Consent is about having the freedom to choose to have sex	No	125 (11.7)	90 (11.1)	35 (13.7)		
	
	Unsure	177 (16.6)	123 (15.2)	54 (21.1)		
	
	Yes	762 (71.6)	595 (73.6)	167 (65.2)	7.01	0.030

Consent needs to be verbally agreed	No	297 (27.8)	201 (24.8)	96 (37.4)		
	
	Unsure	228 (21.4)	176 (21.7)	52 (20.2)		
	
	Yes	542 (50.8)	433 (53.5)	109 (42.4)	15.91	< 0.001

Being drunk affects one's capacity to make reasonable decisions	Disagreee	42 (3.9)	26 (3.2)	16 (6.2)		
	
	Undecided	7 (0.7)	4 (0.5)	3 (1.2)		
	
	Agree	1023 (95.4)	783 (96.3)	240 (92.7)	6.06	0.048

Being drunk affects one's capacity to consent to sex	Disagree	165 (15.4)	105 (12.9)	60 (23.2)		
	
	Undecided	44 (4.1)	34 (4.2)	10 (3.9)		
	
	Agree	862 (80.5)	673 (82.9)	189 (73.0)	15.80	< 0.001

A drunk person is unable to consent to sex	Disagree	755 (70.6)	552 (67.9)	203 (79.0)		
	
	Undecided	130 (12.1)	110 (13.5)	20 (7.8)		
	
	Agree	185 (17.3)	151 (18.6)	34 (13.2)	11.94	0.003

If a person is drunk, as long as they remain physically conscious they will be capable of choosing to have sex	Disagree	716 (66.9)	565 (69.6)	151 (58.5)		
	
	Undecided	136 (12.7)	104 (12.8)	32 (12.4)		
	
	Agree	218 (20.4)	143(17.6)	75 (29.1)	16.22	< 0.001

Person A is mildly drunk; person B is severely drunk and too drunk to give consent. They have sex. Next day B states rape has occurred. Should A be held responsible for rape?	Disagree	291 (27.1)	198(24.3)	93 (35.9)		
	
	Undecided	207 (19.3)	164 (20.1)	43 (16.6)		
	
	Agree	577 (53.7)	454 (55.6)	123 (47.5)	13.52	0.001

Person A is moderately drunk; person B is severely drunk and too drunk to give consent. They have sex. Next day B states rape has occurred. Should A be held responsible for rape?	Disagree	503 (46.8)	353 (43.3)	150 (57.9)		
	
	Undecided	238 (22.1)	199 (24.4)	39 (15.1)		
	
	Agree	334 (31.1)	264 (32.4)	70 (27.0)	18.55	< 0.001

Person A and B are severely drunk: A too drunk to establish if consent is present, B too drunk to consent. They have sex. Next day B states rape. Should A be held responsible for rape?	Disagree	857 (79.7)	637 (78.1)	220 (84.9)		
	
	Undecided	153 (14.2)	132 (16.2)	21 (8.1)		
	
	Agree	65 (6.0)	47 (5.8)	18 (6.9)	10.62	0.005

How would you describe this scenario? Person A and B are severely drunk: A too drunk to establish if consent is present, B too drunk to consent. They have sex. Next day B states rape.	Consensual	149 (13.9)	102 (12.5)	47 (18.4)		
	
	Rape	32 (3.0)	21 (2.6)	11 (4.3)		
	
	Mid-point	720 (67.4)	560 (68.9)	160 (62.5)		
	
	Undecided	168 (15.7)	130 (16.0)	38 (14.8)	7.97	0.047

A significant number of rapes reported to the police are false allegations	Disagree	436 (40.7)	356 (43.7)	80 (31.3)		
	
	Undecided	239 (22.3)	185 (22.7)	54 (21.1)		
	
	Agree	396 (37.0)	274 (33.6)	122 (47.7)	18.00	< 0.001

Being drunk when having sex increases the likelihood of a false rape allegation	Disagree	130 (12.1)	109 (13.4)	21 (8.2)		
	
	Undecided	73 (6.8)	65 (8.0)	8 (3.1)		
	
	Agree	869 (81.1)	641 (78.7)	228 (88.7)	13.59	0.001

Who do you have sex with?	Opposite sex	950 (89.0)	725 (89.5)	225 (87.5)		
	
	Same sex	58 (5.4)	36 (4.4)	22 (8.6)		
	
	Both sexes	59 (5.5)	49 (6.0)	10 (3.9)	7.81	0.020

Only variables which differed significantly by sex are displayed.

**Table 2:** Multivariate comparison of male and female students' alcohol consumption and sexual activity; male predictive factors

Variable	Category	Adjusted odds ratio (95% confidence interval)	Wald	P
Relevance of other person flirting with you when deciding whether they will agree to have sex with you	Irrelevant	Reference	10.24	0.006
	
	Undecided	1.89 (1.15 - 3.11)	6.28	0.012
	
	Relevant	1.83 (1.24 - 2.71)	9.28	0.002

Relevance of the other person removing some of their clothes when deciding whether they will agree to have sex with you	Irrelevant	Reference	9.15	0.010
	
	Undecided	0.82 (0.27 - 2.47)	0.13	0.718
	
	Relevant	2.29 (0.91 - 5.72)	3.13	0.077

Relevance of the other person removing some of your clothes when deciding whether they will agree to have sex with you	Irrelevant	Reference	5.44	0.066
	
	Undecided	3.42 (1.10 - 10.62)	4.52	0.033
	
	Relevant	3.04 (1.15 - 8.00)	5.07	0.024

Consent is about agreeing to sex through choice	No	Reference	8.09	0.018
	
	Unsure	0.32 (0.11 - 0.90)	4.68	0.030
	
	Yes	0.30 (0.13 - 0.69)	8.09	0.004

Consent is about having the freedom to choose to have sex	No	Reference	8.60	0.014
	
	Unsure	1.44 (0.80 - 2.60)	1.48	0.224
	
	Yes	0.78 (0.48 - 1.26)	1.06	0.304

Consent needs to be verbally agreed	No	Reference	6.44	0.040
	
	Unsure	0.71 (0.45 - 1.10)	2.35	0.125
	
	Yes	0.63 (0.44 - 0.90)	6.32	0.012

Being drunk affects one's capacity to consent to sex	Disagree	Reference	9.83	0.007
	
	Undecided	0.47 (0.19 - 1.13)	2.87	0.090
	
	Agree	0.53 (0.35 - 0.79)	9.48	0.002

Person A is moderately drunk; person B is severely drunk and too drunk to give consent. They have sex. Next day B states rape has occurred. Should A be held responsible for rape?	Disagree	Reference	10.18	0.006
	
	Undecided	0.50 (0.32 - 0.79)	9.15	0.002
	
	Agree	0.70 (0.48 - 1.02)	3.43	0.064

A significant number of rapes reported to the police are false allegations	Disagree	Reference	8.61	0.014
	
	Undecided	1.38 (0.89 - 2.14)	2.04	0.153
	
	Agree	1.75 (1.20 - 2.54)	8.61	0.003

Being drunk when having sex increases the likelihood of a false rape allegation	Disagree	Reference	7.30	0.026
	
	Undecided	0.77 (0.29 - 2.02)	0.28	0.597
	
	Agree	1.79 (0.99 - 3.24)	3.71	0.054

All the variables in Table 1 were entered into the model; only variables (or variable strata) significantly related to the dependent variable are retained in the logistic regression model. N = 994. Hosmer and Lemeshow Goodness-of-Fit Test: *X*^2 ^= 4.74, P = 0.79.

Presented with three scenarios depicting sex between two intoxicated people (in two scenarios the level of intoxication was disproportionate), female respondents were more likely than male respondents to state that the scenario depicted rape. Both men and women were more likely to disagree that rape occurred if both parties were equally intoxicated. In the multivariate analysis, where one person was moderately drunk and the other severely so, compared to women, men were more likely to say they were undecided whether it was rape than to disagree (AOR = 0.50, 95% CI = 0.32-0.79). Male respondents were significantly more likely than female respondents to agree that a significant proportion of rapes reported to the police are false allegations (AOR = 1.75, 95% CI = 1.20-2.54). The remaining three variables in the logistic regression model differed significantly between males and females but the 95% CI for the strata level analysis contained the value one (Table 2).

Different variables distinguished whether a female or male student had experienced alcohol-related non-consensual sex since the age of 14 (Tables 3 and 4). A significantly greater proportion of females that had experienced alcohol-related non-consensual sex were hazardous drinkers (82.2%) compared to the females that had not experienced alcohol-related non-consensual sex (62.9%, AOR = 2.82, 95% CI 1.96-4.06). When given a hypothetical scenario where two people that are disproportionately intoxicated have sex and the more severely drunk person claims to have been raped, a greater proportion of women who had experienced alcohol-related non-consensual sex agreed this was rape (AOR = 1.51, 95% CI = 1.03-2.19). Altogether, three quarters of women agreed that being drunk increased the likelihood of a false rape allegation; a significantly smaller proportion of women who had experienced alcohol-related non-consensual sex thought this was the case (AOR = 0.53, 95% CI = 0.35-0.82).

**Table 3:** Bivariate comparison of factors associated with students experiencing alcohol-related non-consensual sex, by sex

Variable	Category	Total	Never experienced alcohol-related non-consensual sex	Experienced alcohol-related non-consensual sex	*X*^2^	P
			**Female students**			
		
		**N (%)**	**N (%)**	**N (%)**		

Alcohol consumption	Non hazardous	244 (30.5)	196 (37.1)	48 (17.8)		
	
	Hazardous	555 (69.5)	333 (62.9)	222 (82.2)	31.30	< 0.001

A is mildly drunk, B severely. B is too drunk to give consent. They have sex. Next day B states rape occurred. Should A be held responsible for rape?	Disagree	197 (24.3)	139 (25.8)	58 (21.3)		
	
	Undecided	161(19.9)	117 (21.7)	44 (16.2)		
	
	Agree	453 (55.9)	283 (52.5)	170 (62.5)	7.50	0.023

A significant number of rapes reported to the police are false allegations	Disagree	353 (43.6)	211 (39.2)	142 (52.2)		
	
	Undecided	184 (22.7)	132 (24.5)	52 (19.1)		
	
	Agree	273 (33.7)	195 (36.2)	78 (28.7)	12.40	0.002

Being drunk when having sex increases the likelihood of a false rape allegation	Disagree	108 (13.3)	58 (10.8)	50 (18.4)		
	
	Undecided	65 (8.0)	42 (7.8)	23 (8.5)		
	
	Agree	637 (78.6)	438 (81.4)	199 (73.2)	9.49	0.009

Women who regret sex when drunk are more likely to report a false rape allegation	Disagree	243 (30.0)	145 (27.0)	98 (36.0)		
	
	Undecided	100 (12.3)	69 (12.8)	31 (11.4)		
	
	Agree	467 (57.7)	324 (60.2)	143 (52.6)	7.10	0.029

Women are more interested in sex when drunk compared to when sober	Disagree	324 (40.1)	215 (40.1)	109 (40.1)		
	
	Undecided	96 (11.9)	76 (14.2)	20 (7.4)		
	
	Agree	388 (48.0)	245 (45.7)	143 (52.6)	8.85	0.012

A woman who has drank alcohol and is drunk, should hold some responsibility for a rape/assault that may then happen	Disagree	497 (61.8)	311 (58.2)	186 (68.9)		
	
	Undecided	60 (7.5)	49 (9.2)	11 (4.1)		
	
	Agree	247 (30.7)	174 (32.6)	73 (27.0)	11.34	0.003

			Male students			

		N (%)	N (%)	N (%)		

Have sex with:	Opposite sex	224 (87.5)	184 (91.5)	40 (72.7)		
	
	Same sex	22 (8.6)	13 (6.5)	9 (16.4)		
	
	Both sexes	10 (3.9)	4 (2.0)	6 (10.9)	15.46	< 0.001

Consent is about having the freedom to choose to have sex	No	35 (13.7)	26 (12.9)	9 (17.0)		
	
	Unsure	54 (21.2)	50 (24.8)	4 (7.5)		
	
	Yes	166 (65.1)	126 (62.4)	40 (75.5)	7.49	0.024

A and B are severely drunk. A is too drunk to establish if consent is present. B is too drunk to consent. They have sex. Next day B states rape occurred. How would you describe this?	Consensual	47 (18.4)	35 (17.5)	12 (21.8)		
	
	Rape	10 (3.9)	10 (5.0)	0 (0.0)		
	
	A mid-point	160 (62.7)	119 (59.5)	41 (74.5)		
	
	Undecided	38 (14.9)	36 (18.0)	2 (3.6)	10.72	0.013

Only variables which differed significantly by experience of alcohol related non-consensual sex are displayed.

**Table 4:** Multivariate comparison of factors associated with students experiencing alcohol-related non-consensual sex, by sex

Variable	Category	Female students			Male students		
		**Adjusted odds ratio (95% confidence interval)**	**Wald**	**P**	**Adjusted odds ratio (95% confidence interval)**	**Wald**	**P**

Alcohol consumption	Non hazardous	Reference	-	-	Not entered into the model		
				
	Hazardous	2.82 (1.96-4.06)	30.85	< 0.001			

A is mildly drunk, B severely. B is too drunk to give consent. They have sex. Next day B states rape occurred. Should A be held responsible for rape?	Disagree	Reference	7.82	0.020	Not entered into the model		
				
	Undecided	0.93 (0.58-1.50)	0.08	0.774			
				
	Agree	1.51 (1.03-2.19)	4.54	0.033			

Being drunk when having sex increases the likelihood of a false rape allegation	Disagree	Reference	8.67	0.013	Not entered into the model		
				
	Undecided	0.70 (0.36-1.36)	1.12	0.290			
				
	Agree	0.53 (0.35-0.82)	8.34	0.004			

Have sex with:	Opposite sex	Not entered into the model			Reference	10.26	0.006
					
	Same sex				2.80 (1.07-7.32)	4.39	0.036
					
	Both sexes				6.60 (1.60-27.17)	6.83	0.009

Consent is about having the freedom to choose to have sex	No	Not entered into the model			Reference	6.79	0.034
					
	Unsure				0.20 (0.05-0.76)	5.57	0.018
					
	Yes				0.84 (0.34-2.05)	0.15	0.696

A and B are severely drunk. A is too drunk to establish if consents present. B is too drunk to consent. Both have sex. Next day B states rape occurred. Should A be held responsible for rape? How would you describe this?	Consensual	Not entered into the model			Reference	5.69	0.128
					
	Rape				0	0	0.999
					
	A mid-point				0.99 (0.45-2.19)	0	0.986
					
	Undecided				0.08 (0.01-0.70)	5.26	0.022

Only variables (or variable strata) significantly related to the dependent variable are retained in the logistic regression model. N = 789 for the female logistic regression model. Hosmer and Lemeshow Goodness-of-Fit Test: *X*^2 ^= 1.18, P = 0.95. N = 250 for the male logistic regression model. Hosmer and Lemeshow Goodness-of-Fit Test: *X*^2 ^= 1.70, P = 0.95.

Compared to men who had sex with women only, a greater proportion of men who had sex with other men had experienced alcohol-related non-consensual sex (AOR = 2.80, 95% CI = 1.07-7.32), as had a greater proportion of men who had sex with both men and women (AOR = 6.60, 95% CI = 1.60-27.17) although some cell counts were small. A smaller proportion of men who had experienced alcohol-related non-consensual sex were unsure that consent is about having the freedom to choose to have sex (AOR = 0.20, 95% CI = 0.05-0.76). Finally, when presented with a hypothetical scenario involving two disproportionately drunk people who have sex, a greater proportion of men who had experienced alcohol-related non-consensual sex were undecided around the classification of the intercourse compared to the proportion who deemed it consensual (AOR = 0.08, 95% CI = 0.01-0.70).

## Discussion

Despite amendments to English and Welsh law rape conviction rates remain low [8-11]. Low conviction rates are particularly evident when the complainant is voluntarily intoxicated by alcohol [2,12]; a situation of some concern in light of Police data that show the complainant has been drinking alcohol in approximately half of all rape cases [13]. Here we show that a third of participants had experienced alcohol-related non-consensual sex, demonstrating the significance of this as a public health issue.

Differences between men and women in relation to attitudes and knowledge of sexual events are likely to influence the outcome of dating situations where sex is possible and we show that differences between male and female students exist. While the majority of participants correctly stated that consent involved agreeing to engage in sex through choice, male respondents were less likely to know this or were unsure whether this was reflected in law. Approximately half of respondents incorrectly thought that consent needed to be verbally agreed, with this belief being more likely among women. Men were also less likely to believe that being drunk affects one's capacity to consent to sex. These differences between men and women may result in situations where drunken non-consensual sex is perceived to be consensual by the man.

Male and female students also differed in their attitudes towards the cues that they would deem relevant or informative when deciding whether or not a person wanted to have sex with them. The cues which differed significantly in the multivariate comparison of men and women were the relevance of the other person flirting, and the relevance of the other person removing their own, or the respondent's, clothing (Table 1 and 2). However, a greater proportion of men than women deemed all the cues described in the questionnaire as relevant. This is important given that the effects of alcohol arise from both alcohol's impairment of perception and the nature of the environmental cue [19]. Alcohol intoxication disrupts information processing skills and impairs cognitive processes (so called 'executive functioning') which are important for control of behaviour [39]. In addition, an intoxicated person pays attention to fewer environmental cues, while intoxication also reduces the ability to process the meaning of these cues. As a result, immediate experiences may have a disproportionate influence over behaviour and emotion [19]. If a man perceives that the other person is willing to engage in sex, for example they have been flirting, alcohol-related cognitive disruption may result in them focusing on the prominence of sexual arousal at the expense of less salient cues such as their partner's protests [40]. In such a situation, alcohol induced disinhibition, coupled with a reduction in self-appraisal and a focus on arousal in response to supposedly encouraging behaviour, have the potential to create a situation where pressure or force is used to obtain sex [41,42]. If the parties do not know each other well, it is possible that supposedly encouraging cues will be deemed even more relevant in negotiating the potential for sex.

When presented with three scenarios depicting sex between two intoxicated people the differential level of intoxication between the two parties clearly influenced whether or not a respondent would label the sex as rape (Tables 1 and 2). Both male and female respondents were more likely to label the scenario as rape when the difference in levels of intoxication between the two parties was greater. These findings align with previous research showing that people are reluctant to label a situation as rape when both parties are equally intoxicated [43-45]. We can hypothesise that respondents felt that the impact of alcohol on cognitive functioning could result in a defendant genuinely believing that consent was present even if it was not. In the eyes of the law, alcohol intoxication is not a defence to a charge of rape, yet it may be suggested that respondents viewed comparable drunkenness as a factor that was sufficient to mitigate the defendant's responsibility for ensuring consent. These findings occur against a backdrop of opinion that women who consume alcohol have more culpability if they are a victim of a sexual assault than women who do not drink [9,28,43,46-48]. Taken together such findings support a drinking double standard whereby women are blamed more for a sexual offence when they have consumed alcohol whilst drinking defendants are viewed as less likely to have perpetrated a crime [43-45]. Consequently, societal attitudes around alcohol and culpability appear to work in favour of the defendant but against a complainant. Furthermore, just over a third of respondents in the questionnaire stated that a significant number of rapes reported to the Police are false allegations and that false allegations are more likely when people are drunk; beliefs which were more prominent among men (Tables 1 and 2). The premise that women frequently make false allegations of rape is not supported by recent evidence [13,49,50] and together these findings highlight the biases that may impact in rape cases.

Levels of current drinking were higher among women who had experienced alcohol-related non-consensual sex (Tables 3 and 4) supporting previous findings that demonstrate a link between the amount of alcohol consumed in night time environments and experiencing sexual molestation [51]. While the cross-sectional nature of the current study makes it impossible to determine causality or identify the level of drinking at the time of the alleged incident, there is good evidence to show a relationship between alcohol consumption and being a victim of a sexual assault [1,28,52-54]. Here, women who had experienced alcohol-related non-consensual sex were more likely to label a hypothetical sexual scenario between two people who were disproportionately intoxicated as rape than women who had not experienced alcohol-related non-consensual sex, perhaps because they felt their own experiences were reflected within this hypothetical depiction (Table 3). These women were also less likely to believe that being drunk increased the likelihood of a false rape allegation, again a perception potentially borne from experience.

The questionnaire identified 55 men who had experienced alcohol-related non-consensual sex demonstrating that sexual assault victimisation is not exclusively the domain of women. The small body of research which focuses on men as victims of sexual victimisation shows that men experience this form of violence from both men and women [55,56]. Reporting of non-consensual sex by men is typically inhibited by stigma and stereotypes which often results in the extent of men's non-consensual sexual encounters being under-reported [57]. Here, compared to men who had sex with women, a greater proportion of men who had sex with other men in both homosexual and bisexual relationships had experienced alcohol-related non-consensual sex. Men who had experienced alcohol-related non-consensual sex were less likely to know that consent is about having the freedom to choose to have sex. They were also less likely to describe a situation where both parties are equally intoxicated and have sex as rape, or were undecided whether this was the case (Table 4). Further research on the victimisation of men is needed to help develop an understanding of these factors and elucidate their relevance to homosexual and bisexual men's lives.

Our study has several limitations. The cross sectional design means that it is not possible to ascertain a causal relationship between hazardous drinking and experiencing alcohol-related non-consensual sex; it is possible that experiencing alcohol-related non-consensual sex resulted in increasing drinking levels. The study also relies on retrospective self-reported data. We used non-probability sampling to recruit our participants and are therefore not able to infer whether our results can be generalised to the wider student population. Furthermore, the questionnaire included experiences that could have occurred before being a student. It is therefore not possible to use the results presented here as a measure of the prevalence of alcohol-related non-consensual sex among students. Finally, while the SES questions did provide definitions of alcohol-related non-consensual sex, we did not define what we meant by 'sex' throughout the remaining questions.

## Conclusion

From over a thousand respondents, almost a third said they had experienced alcohol-related non-consensual sex. This demonstrates the role that alcohol plays in coerced sexual activity. Differences between men and women in their understanding of the law, their perceptions of the relevance of sexual cues and their understanding of the impact of alcohol on one's ability to give informed consent may go some way towards explaining how alcohol-related sexual 'misunderstandings' may turn a potentially consensual situation into an act which constitutes a sexual offence. In particular, women were more likely to incorrectly believe that consent needed to be verbalised. The results detailed here support research conducted in the USA demonstrating a link between alcohol use and coerced sexual activity; a link which is likely to exist in other countries given that globally students are high consumers of alcohol [58].

In response, there should be emphasis on promoting messages that focus on the use of alcohol-related strategies to procure non-consensual sex rather than surreptitious drug administration that has grabbed media attention but appears to occur much less frequently [59]. Here we show that both men and women are victims of alcohol-related non-consensual sexual experiences suggesting that organisations that aim to raise awareness of alcohol-related non-consensual sex should focus upon both genders as victims. In light of the misunderstandings highlighted here in relation to the law of consent, its parameters and whether it must be verbalised to be deemed legal, further dissemination of messages around rape and the legal stance is necessary in order to make clear what is acceptable and unacceptable sexual behaviour. In particular, messages are necessary that emphasise that alcohol intoxication is not a defence to a charge of rape and that the consequences of not actively establishing whether consent is given could be significant if charged and found guilty. Educational messages must also challenge the inaccurate belief that a significant proportion of rape allegations are false by providing factual information that can dispel such myths. While there is evidence from the USA that school-based educational programmes can be used to reduce dating violence among adolescents [60,61], in the UK any such initiatives are set against a backdrop where preloading (drinking before going out), binge drinking and drunkenness are the norm for a significant section of society [51,62], and where current national drinking guidelines could be interpreted to suggest infrequent drunkenness is acceptable [63].

## Competing interests

MB has acted as an adviser to DrinkAware in the previous three years.

## Authors' contributions

CG, AC and CB designed the study. CG managed the literature searches and all authors related findings to previous research. CG and CB conducted the statistical analyses. All authors contributed to and approved the final manuscript.

## Pre-publication history

The pre-publication history for this paper can be accessed here:

http://www.biomedcentral.com/1471-2458/12/216/prepub
